# Long-term phase 3 study of esaxerenone as mono or combination therapy with other antihypertensive drugs in patients with essential hypertension

**DOI:** 10.1038/s41440-019-0314-7

**Published:** 2019-09-25

**Authors:** Hiromi Rakugi, Sadayoshi Ito, Hiroshi Itoh, Yasuyuki Okuda, Satoru Yamakawa

**Affiliations:** 1 Department of Geriatric and General Medicine, Osaka University Graduate School of Medicine, 2-2 Yamadaoka, Suita, Osaka 565-0871 Japan; 2 Division of Nephrology, Endocrinology and Vascular Medicine, Department of Medicine, Tohoku University School of Medicine, 2-1 Seiryo-machi, Aoba, Sendai, Miyagi 980-8575 Japan; 3 Department of Endocrinology, Metabolism and Nephrology, Keio University, School of Medicine, 35 Shinanomachi, Shinjuku, Tokyo 160-8582 Japan; 4 Daiichi Sankyo Co., Ltd., 1-2-58 Hiromachi, Shinagawa, Tokyo 140-8710 Japan

**Keywords:** Antihypertensive agents, Combination drug therapy, Eesaxerenone, Essential hypertension, Japan

## Abstract

This study investigated the long-term antihypertensive effects of esaxerenone, a novel nonsteroidal mineralocorticoid receptor blocker, alone or in combination with a calcium channel blocker (CCB) or a renin–angiotensin system (RAS) inhibitor, in Japanese patients with essential hypertension. Patients were treated with esaxerenone starting at 2.5 mg/day increasing to 5 mg/day if required to achieve blood pressure (BP) targets as a monotherapy or with a CCB or RAS inhibitor. After the first 12 weeks of treatment, an additional antihypertensive agent could be added if required to achieve the target BP; the total treatment period was 28 or 52 weeks. The primary endpoint was a change from baseline in sitting BP. Of the 368 enrolled patients, 245 received monotherapy, and 59 and 64, respectively, took a CCB or RAS inhibitor concurrently. Mean changes from baseline in sitting systolic/diastolic BP (95% confidence intervals) at weeks 12, 28 and 52 were −16.1 (−17.3, −14.9)/−7.7 (−8.4, −6.9), −18.9 (−20.2, −17.7)/−9.9 (−10.7, −9.2), and −23.1 (−25.0, −21.1)/−12.5 (−13.6, −11.3) mmHg, respectively (all *P* < 0.0001 vs baseline). Similar BP reductions at these weeks were observed between all patient subgroups stratified by age, and the observed decreases in 24-h ambulatory BP were consistent with the efficacy observed in sitting BP. Esaxerenone was also well-tolerated with a rate of hyperkalemia at 5.4% (serum potassium ≥5.5 mEq/L), indicating a good safety profile for treatment over the long-term or in combination with a CCB or RAS inhibitor. In conclusion, esaxerenone may be a promising treatment option for patients with hypertension.

## Introduction

Aldosterone acts on mineralocorticoid receptors (MRs) on renal tubular epithelial cells to regulate electrolyte levels and blood fluid volume by promoting sodium reabsorption and urinary potassium (K^+^) excretion. Therefore, increasing aldosterone levels have been associated with an increased incidence and severity of hypertension [1–5]. Additionally, aldosterone has been linked with the development and progression of a number of comorbidities associated with hypertension, including endothelial dysfunction, left ventricular hypertrophy, chronic kidney disease, heart failure, stroke, and obstructive sleep apnea [6–14]. As a result, agents that block the action of aldosterone at MRs have therapeutic activity in a number of conditions, such as hypertension, heart failure, and microalbuminuria [15–23].

Existing MR blockers, including spironolactone and eplerenone, have therapeutic utility as add-on therapies for treatment-resistant hypertension [24, 25]. However, the use of these agents is associated with adverse drug reactions that limit their usefulness in clinical practice. Spironolactone has low MR-binding specificity, leading to treatment-related adverse effects, such as sex hormone-related events [26–28]. Eplerenone has higher MR-binding specificity than was observed for spironolactone, but is still contraindicated in some patients with renal dysfunction [29–31].

Esaxerenone is a novel nonsteroidal oral MR blocker with BP-lowering activity [32, 33]. Recently, published phase 1 and phase 2 clinical studies have shown that esaxerenone effectively lowers BP and is well-tolerated [33–35]. However, there are currently no data on the longer-term efficacy and safety of esaxerenone.

Calcium channel blockers (CCBs) and renin–angiotensin system (RAS) inhibitors are recommended as first-line treatment options for patients with hypertension according to the Japanese Society of Hypertension guidelines (JSH2014) [36]. MR blockers are often recommended as an add-on therapy [25, 36, 37] and are likely to be coadministered with CCBs or RAS inhibitors as second- or third-line treatments. The combination of eplerenone with RAS inhibitors has recently been suggested as a potential treatment option in patients with hypertension and chronic kidney disease, especially in whom salt intake is high [38].

To generate evidence relevant to the usage of esaxerenone in current clinical settings, this phase 3 clinical study investigated the antihypertensive effects of esaxerenone either as a monotherapy or in combination with a CCB or RAS inhibitor and administered for 28 or 52 weeks in Japanese patients with essential hypertension.

## Methods

### Study design

This multicenter, open-label, optional dosage escalation, long-term phase 3 clinical study was conducted at 19 centers in Japan between March 2016 and July 2017. The study protocol was approved by the relevant institutional review boards. All study procedures were carried out in accordance with the guidelines stated in the Declaration of Helsinki and Good Clinical Practice, and all patients provided written informed consent prior to enrollment in the study.

There was a 4-week observation period at baseline followed by two treatment periods: the first from baseline to week 12 and the second from week 12 until week 28 or week 52 (Supplementary Fig. 1). In the first treatment period, eligible patients received esaxerenone monotherapy or esaxerenone in combination with a CCB or RAS inhibitor. During the second treatment period, the dosage of existing antihypertensives could be increased or an additional antihypertensive agent could be added as required to achieve the target BP.

### Patients

Eligible patients were ≥20 years old and had previously untreated essential hypertension or had received only one RAS inhibitor or CCB as a baseline antihypertensive agent at the start of the observation period. All patients had a sitting systolic BP (SBP) of 140 to <180 mmHg and a sitting diastolic BP (DBP) of 90 to <110 mmHg, a 24-h ambulatory BP of ≥130/80 mmHg, and an estimated glomerular filtration rate (eGFR) of ≥60 mL/min/1.73 m^2^. Key exclusion criteria were secondary hypertension (e.g., renovascular hypertension), orthostatic hypotension, cardiovascular disease or intervention within the previous 6 months, cerebrovascular disease within the previous year, serum K^+^ level <3.5 or ≥5.1 mEq/L (or ≥4.8 mEq/L in patients also receiving a RAS inhibitor), HbA1c (measured using the National Glycohemoglobin Standardization Program) ≥8.4%, type 1 diabetes, and type 2 diabetes with diabetic nephropathy or albuminuria. Patients were withdrawn from the study if significant adverse events occurred or in the event of serious violations of guidelines or the study protocol.

### Treatment

Patients who had not received any antihypertensive agents or were treated with agents other than a RAS inhibitor or CCB at enrollment were allocated to the monotherapy group. Those who were receiving one RAS inhibitor or CCB with or without other classes of antihypertensive agents at enrollment were allocated to the combination therapy group. During the observation period, there was a 4-week washout period for prior antihypertensive therapy in the monotherapy group and for prior antihypertensive agents other than a RAS inhibitor or CCB in the combination group.

Patients in the monotherapy group received esaxerenone alone during the first 12-week treatment period, while those in the combination therapy group received esaxerenone in combination with either a CCB or a RAS inhibitor. Esaxerenone therapy was initiated at a dosage of 2.5 mg/day based on the results of a previous dosage-finding study [35]. If the SBP remained ≥140 mmHg or DBP remained ≥90 mmHg (or SBP ≥130 mmHg or DBP ≥80 mmHg in patients with diabetes) at weeks 4, 6, or 8, the esaxerenone dosage was increased to 5 mg/day and was maintained at this level until week 12. The criteria for increasing the esaxerenone dosage also included a serum K^+^ level <5.1 mEq/L (or <4.8 mEq/L in patients also receiving a RAS inhibitor). During the first treatment period, no reductions in the esaxerenone dosage were permitted, and the dosage of the concomitant RAS inhibitor or CCB was fixed. In the second treatment period (after week 12), the esaxerenone dosage at week 12 was continued until week 28 or 52 (without any planned change in dosage). For patients whose BP was not sufficiently controlled after week 12, one additional antihypertensive drug administration (CCB, RAS inhibitor, or thiazide diuretic) was permitted in the monotherapy group, and dose escalation of the baseline CCB, RAS inhibitor, or one additional concomitant antihypertensive drug (other CCB, RAS inhibitor, or thiazide diuretic) was permitted in the combination therapy group. The use of fixed-dose combinations was not allowed.

### Endpoints

The primary efficacy endpoints were the change from baseline in sitting SBP and DBP after 12, 28, and 52 weeks of treatment. The secondary efficacy endpoint was the change from baseline in 24-h BP as determined using ambulatory BP monitoring at weeks 12, 28, and 52. Other efficacy endpoints included the proportion of patients who achieved target 24-h BP (<140/90 mmHg) and changes in BP for patient subgroups based on age (<65 vs ≥65 years old), baseline SBP (<160 vs ≥160 mmHg), and the presence or absence of diabetes. Additional measurements included the plasma aldosterone concentration (PAC), plasma renin activity (PRA), and the levels of human atrial natriuretic peptide (hANP) and N-terminal pro B-type natriuretic peptide (NT-proBNP).

Safety endpoints included the incidence of adverse events, laboratory tests, vital signs, the proportion of patients with serum K^+^ levels ≥5.5 mEq/L, the proportion of patients with serum K^+^ levels ≥6.0 mEq/L or ≥5.5 mEq/L on two consecutive measurements, and the overall tolerability of study treatments.

### Assessments

Sitting BP was measured at week 3 and at the end of the observation period. During the treatment period, sitting BP was measured every 2 weeks until week 12 and every 4 weeks until week 28 or 52 using an automatic BP monitor (HEM-759P Fuzzy device, Omron Healthcare Co., Ltd., Kyoto, Japan). At each assessment, BP measurements were repeated three times; the mean value was used for decisions about dose escalation or add-on therapy and discontinuation. Follow-up observations were performed 1 week after the end of each treatment period or at the completion or discontinuation of the study drug.

Additionally, 24-h BP was measured at week 3 of the observation period and weeks 12, 28, and 52 of the treatment period using an ambulatory BP monitor (TM-2433, A & D Co., Ltd., Tokyo, Japan). BP measurements were taken over a period of at least 25 h at 30-min intervals.

PAC was measured using a radioimmunoassay, and PRA was measured using an enzyme immune assay on blood samples collected during weeks 12, 28, and 52 using previously described methods [33].

### Statistical analysis

Based on guidance from the International Council for Harmonisation for efficacy studies [39], the numbers of patients completing 28 and 52 weeks of esaxerenone treatment were set at 300 and 100, respectively. Taking into account dropouts, the required number of patients was set at 360, including 60 who received esaxerenone in combination with a CCB and 60 who received esaxerenone and a RAS inhibitor.

The full analysis set (FAS) included all patients who had provided informed consent, met the inclusion criteria, received the study drug at least once, and had efficacy endpoint data measured at least once during the treatment period. The safety analysis set included all patients who provided informed consent except for those who did not receive any doses of the study drug.

For sitting and 24-h ambulatory BP (SBP and DBP), point estimates of differences between measurements obtained at baseline and weeks 12, 28, and 52 were calculated along with their 95% confidence interval (CI) values and compared using paired *t*-tests. The last observation carried forward method was applied for missing BP data. The proportion of patients who achieved the target BP was assessed using the point estimate and corresponding exact 95% CI. The BP endpoint was also assessed in patient subgroups. Summary statistics were calculated for PAC, PRA, hANP, and NT-proBNP measured at each timepoint and for changes from baseline. All statistical analyses were conducted using SAS System Version 9.3 (SAS Institute Japan Ltd., Tokyo, Japan).

## Results

### Patients

**Fig. 1:**
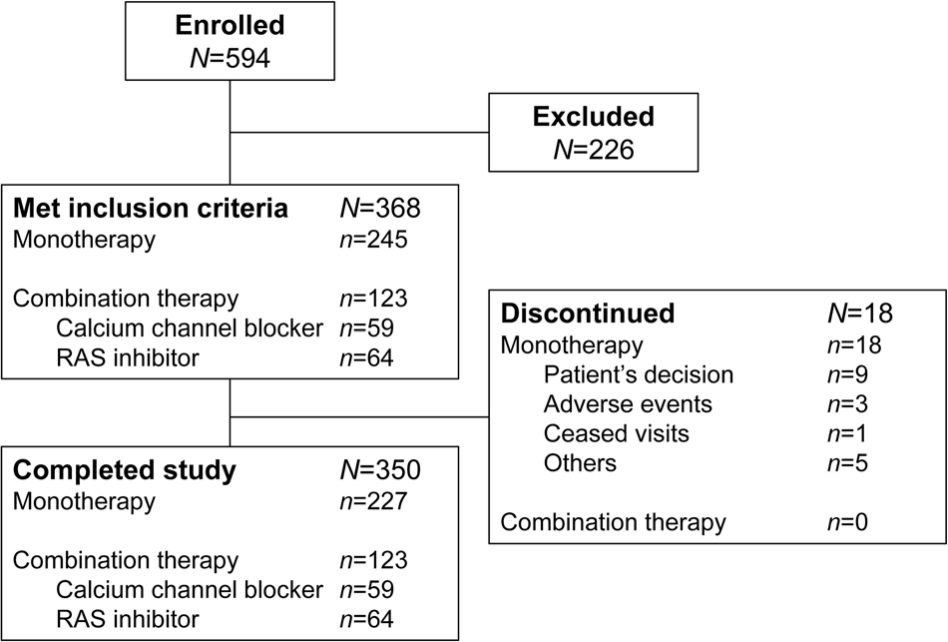
Patient disposition. RAS renin–angiotensin system

Of the 594 patients who provided informed consent, 368 met the inclusion criteria and were enrolled in the study (Fig. 1). Of these, 59 received a concomitant CCB, 64 received a RAS inhibitor, and 245 received esaxerenone monotherapy. A total of 350 patients completed the study, but all 368 patients were included in the FAS and safety analysis set. The mean ± standard deviation (SD) age of all patients was 56.2 ± 9.2 years old, and 77.7% were male (Table 1).

**Table 1:** Baseline demographic and clinical characteristics

		Esaxerenone		
	Total (*N* = 368)	Monotherapy (*n* = 245)	+ CCB (*n* = 59)	+ RAS inhibitor (*n* = 64)
Male, *n* (%)	286 (77.7)	186 (75.9)	52 (88.1)	48 (75.0)
Age, years	56.2 ± 9.2	55.9 ± 9.4	56.1 ± 8.9	57.2 ± 8.9
Age ≥65 years, *n* (%)	78 (21.2)	51 (20.8)	13 (22.0)	14 (21.9)
Weight, kg	71.3 ± 12.3	70.3 ± 11.9	77.2 ± 12.8	70.0 ± 11.7
Body mass index, kg/m^2^	25.7 ± 3.6	25.4 ± 3.5	27.2 ± 4.2	25.6 ± 3.2
SBP, mmHg	155.2 ± 9.6	155.4 ± 10.0	154.2 ± 9.2	155.2 ± 8.6
DBP, mmHg	97.9 ± 5.3	97.5 ± 5.1	97.8 ± 5.1	99.8 ± 5.7
24-h average ambulatory SBP, mmHg	159.0 ± 14.1	160.0 ± 14.3	156.8 ± 13.6	157.1 ± 13.7
24-h average ambulatory DBP, mmHg	95.5 ± 7.7	95.7 ± 7.7	93.9 ± 7.3	96.5 ± 7.9
Hypertension grade, *n* (%)				
Grade I	176 (47.8)	123 (50.2)	31 (52.5)	22 (34.4)
Grade II	192 (52.2)	122 (49.8)	28 (47.5)	42 (65.6)
Prior treatment for hypertension, *n* (%)^a^	244 (66.3)	121 (49.4)	59 (100.0)	64 (100.0)
Diabetes, *n* (%)	67 (18.2)	55 (22.4)	6 (10.2)	6 (9.4)
Serum K^+^, mEq/L	4.17 ± 0.27	4.18 ± 0.27	4.15 ± 0.26	4.16 ± 0.29
Serum K^+^ ≥4.5 mEq/L, *n* (%)	58 (15.8)	37 (15.1)	9 (15.3)	12 (18.8)
eGFR, mL/min/1.73 m^2^	79.6 ± 12.7	79.2 ± 13.1	82.7 ± 13.3	78.4 ± 10.0
HbA1c, %	5.78 ± 0.61	5.81 ± 0.68	5.76 ± 0.49	5.67 ± 0.43

Values are mean ± standard deviation, or number of patients (%)

*CCB* calcium channel blocker, *DBP* diastolic blood pressure, *eGFR* estimated glomerular filtration rate, *HbA1c* glycated hemoglobin, *RAS* renin–angiotensin system, *SBP* systolic blood pressure

^a^Within 4 weeks prior to the run-in period

### Treatment

Overall, 368 patients were treated for 28 weeks, and 147 were treated for 52 weeks. All patients started esaxerenone at a dosage of 2.5 mg/day, and by week 12, this had been increased to 5 mg/day in 64.1% (*n* *=* 157/245) of the patients receiving monotherapy, 67.8% (*n* *=* 40/59) of those receiving combination therapy with a CCB, and 56.3% (*n* *=* 36/64) of those receiving combination therapy with a RAS inhibitor. The corresponding proportions of patients who had an add-on antihypertensive therapy after week 12 were 36.3%, 16.9%, and 25.0%, respectively (Table 2).

**Table 2:** Esaxerenone treatment and use of add-on antihypertensives from week 12 onwards (full analysis set)

	Esaxerenone			
	Monotherapy (*n* = 245)	+ CCB (*n* = 59)	+ RAS inhibitor (*n* = 64)	Total (*N* = 368)
Duration groups of esaxerenone treatment, *n* (%)^a^				
28 weeks group	143 (58.4)	35 (59.3)	43 (67.2)	221 (60.1)
52 weeks group	102 (41.6)	24 (40.7)	21 (32.8)	147 (39.9)
Esaxerenone dosage at week 12, *n* (%)				
2.5 mg/day	82 (33.5)	19 (32.2)	28 (43.8)	129 (35.1)
5 mg/day	157 (64.1)	40 (67.8)	36 (56.3)	233 (63.3)
Esaxerenone dosage at last treatment, *n* (%)				
2.5 mg/day	62 (25.3)	17 (28.8)	25 (39.1)	104 (28.3)
5 mg/day	183 (74.7)	42 (71.2)	39 (60.9)	264 (71.7)
Add-on antihypertensive drug from week 12^b^, *n* (%)	89 (36.3)	10 (16.9)	16 (25.0)	115 (31.3)
CCB	76 (31.0)	1 (1.7)	16 (25.0)	93 (25.3)
Thiazide diuretic	1 (0.4)	0 (0.0)	0 (0.0)	1 (0.3)
RAS inhibitor	9 (3.7)	9 (15.3)	0 (0.0)	18 (4.9)
Other	3 (1.2)	0 (0.0)	0 (0.0)	3 (0.8)

*CCB* calcium channel blocker, *RAS* renin–angiotensin system

^a^All 368 patients received esaxerenone until week 28

^b^Except for the baseline CCB or RAS inhibitor

### Efficacy

**Fig. 2:**
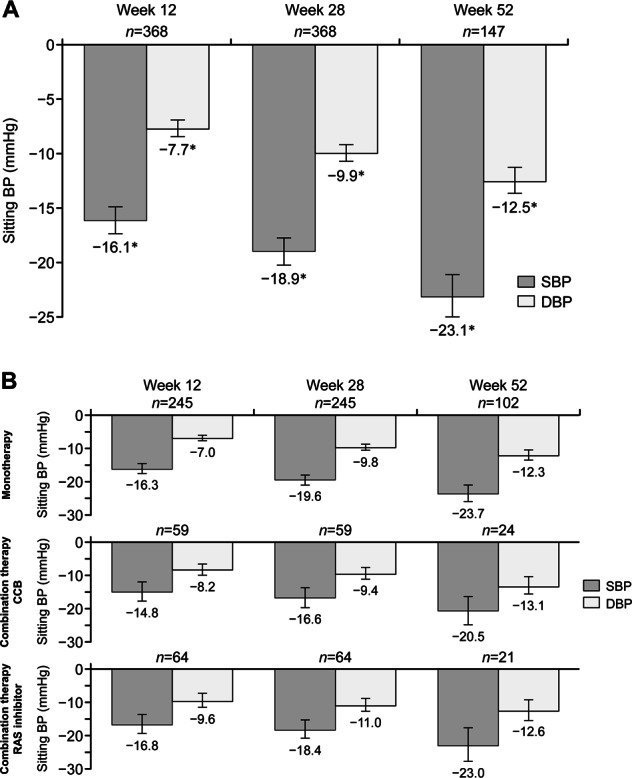
Mean change from baseline in sitting BP (SBP/DBP) for each treatment group: **a** all patients and **b** monotherapy and combination therapy (full analysis set). Data are shown as the mean (95% confidence interval); paired *t*-test. *P < 0.0001 vs baseline. BP blood pressure, CCB calcium channel blocker, DBP diastolic BP, RAS renin–angiotensin system, SBP systolic BP

**Fig. 3:**
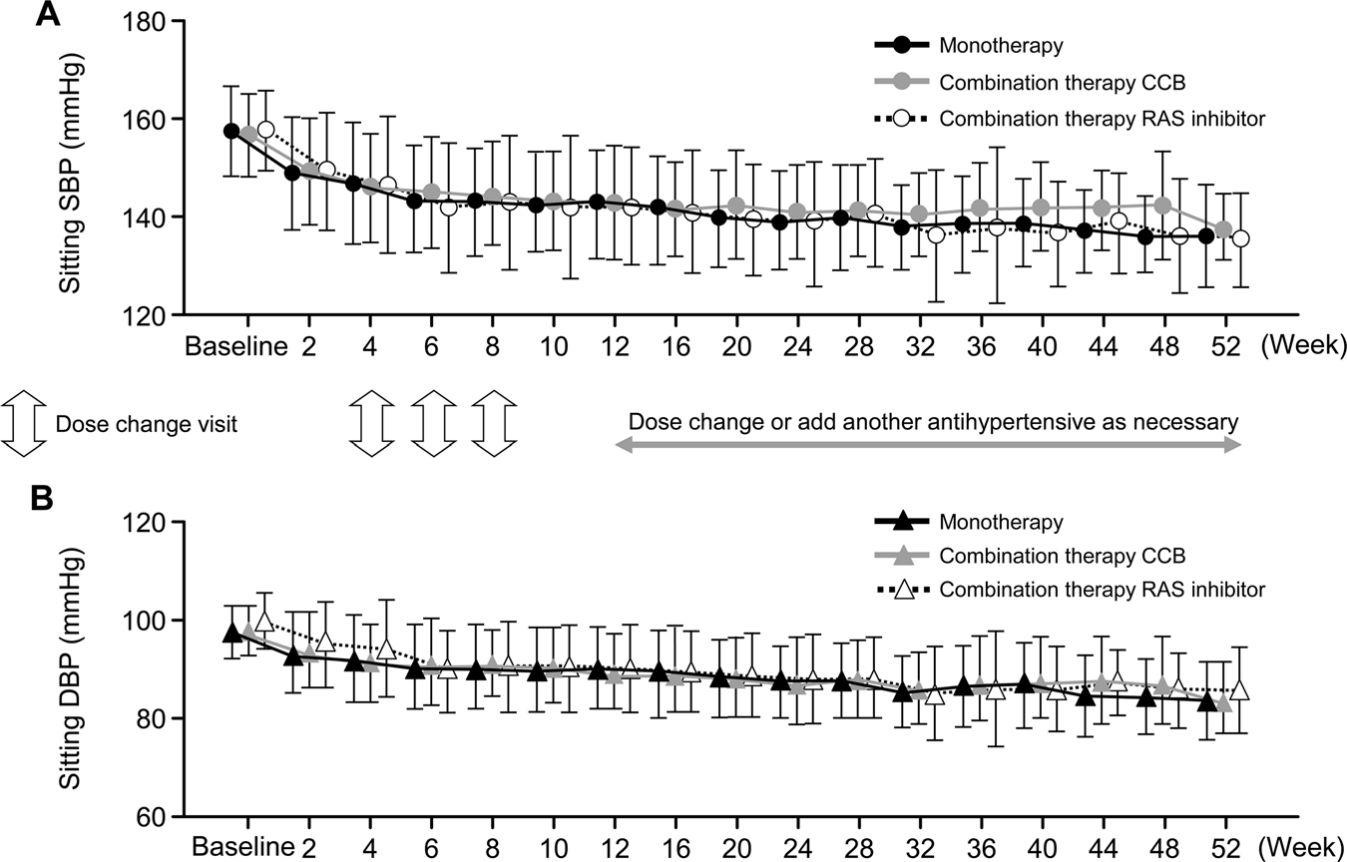
Mean change from baseline in sitting SBP (**a**) and DBP (**b**) over 52 weeks (full analysis set). Data are shown as the mean ± SD. CCB calcium channel blocker, DBP diastolic blood pressure, RAS renin–angiotensin system, SBP systolic blood pressure

The overall mean changes in sitting SBP/DBP (95% CI) between baseline and weeks 12, 28, and 52 were −16.1 (−17.3, −14.9)/−7.7 (−8.4, −6.9) mmHg (*n* = 368), −18.9 (−20.2, −17.7)/−9.9 (−10.7, −9.2) mmHg (*n* = 368), and −23.1 (−25.0, −21.1)/−12.5 (−13.6, −11.3) mmHg (*n* = 147), respectively (all *P* < 0.0001 vs baseline) (Fig. 2a). Reductions in BP were similar across all treatment groups. Patients who received esaxerenone monotherapy had mean SBP/DBP changes (95% CI) of −16.3 (−17.7, −14.8)/−7.0 (−7.9, −6.1) mmHg from baseline to week 12 and −23.7 (−26.2, −21.2)/−12.3 (−13.7, −10.8) mmHg from baseline to week 52 (Fig. 2b). In patients receiving esaxerenone in combination with a CCB, the mean changes (95% CI) in SBP/DBP were −14.8 (−17.8, −11.9)/−8.2 (−9.8, −6.5) mmHg at week 12 and −20.5 (−24.8, −16.2)/−13.1 (−15.7, −10.5) at week 52 (Fig. 2b). The corresponding values in patients receiving esaxerenone plus a RAS inhibitor were −16.8 (−19.8, −13.9)/−9.6 (−11.8, −7.5) mmHg and −23.0 (−28.0, −17.9)/−12.6 (−15.7, −9.5) mmHg, respectively (Fig. 2b). Changes in sitting SBP and DBP were sustained throughout the treatment period and were similar across the three treatment groups (Figs. 2b and 3). Changes in sitting SBP and DBP in patients who received esaxerenone monotherapy without any additional antihypertensive medications after week 12 were similar to those observed in the other treatment groups (Supplementary Table 1).

For the 24-h ambulatory SBP/DBP, the overall mean changes (95% CI) from baseline to weeks 12, 28, and 52 were −11.8 (−13.3, −10.3)/−5.9 (−6.7, −5.2) mmHg (*n* = 354), −13.2 (−14.9, −11.5)/−7.3 (−8.1, −6.5) mmHg (*n* = 347), and −17.9 (−20.6, −15.2)/−9.2 (−10.5, −8.0) mmHg (*n* = 133), respectively (all *P* < 0.0001 vs baseline) (Supplementary Fig. 2A). Changes in 24-h BP were also similar across all the treatment groups (Supplementary Fig. 2B). The proportions of patients who had achieved target sitting BP (SBP/DBP <140/90 mmHg) at the end of treatment were 67.6%, 62.5%, and 57.1% in the esaxerenone monotherapy, esaxerenone with CCB, and esaxerenone with RAS inhibitor groups, respectively (Supplementary Fig. 3).

Reductions in sitting BP during an antihypertensive therapy were generally similar in patient subgroups based on age and the presence or absence of diabetes, although patients with a higher baseline SBP and diabetes tended to have greater reductions in SBP during an antihypertensive therapy (Supplementary Table 2).

### Additional endpoints

Small increases in PAC between baseline and later timepoints were observed in all treatment groups, with slightly greater increases observed in the esaxerenone monotherapy and CCB combination groups than in the RAS inhibitor combination group (Supplementary Fig. 4). PRA also increased from baseline during esaxerenone treatment, with similar increases observed across all treatment groups (Supplementary Fig. 5). Reductions in the levels of hANP and NT-proBNP were observed in all treatment groups. Decreases in natriuretic peptide levels in the CCB combination group were numerically smaller than those in the other two groups, probably due to lower baseline levels observed in the CCB combination group (Supplementary Fig. 6). The levels of NT-proBNP tended to decrease over the entire 52-week treatment period (Supplementary Fig. 6).

### Safety

The overall incidence of treatment-emergent adverse events was 68.8%, while the individual incidences were 65.3%, 78.0%, and 73.4% in the esaxerenone monotherapy, CCB combination, and RAS inhibitor combination groups, respectively (Table 3). The corresponding rates for adverse drug reactions were 18.4%, 23.7%, and 18.8%, respectively. There were no obvious differences in the incidences of treatment-emergent adverse events or adverse drug reactions between the esaxerenone monotherapy and combination therapy groups. The mean ± SD baseline serum K^+^ level for all patients was 4.17 ± 0.27 mEq/L (Table 1), and although a slight increase in serum K^+^ levels (0.2–0.3 mEq/L; data not shown) was observed after 2 weeks, these levels remained stable throughout the study at weeks 12, 28, and 52, with mean ± SD differences of 0.06 ± 0.30, 0.12 ± 0.34, and 0.03 ± 0.31 mEq/L, respectively. The changes in serum K^+^ levels were similar across all treatment groups. The overall mean ± SD eGFR was 79.6 ± 12.7 mL/min/1.73 m^2^ at baseline (Table 1) and 74.5 ± 13.8, 75.0 ± 13.2, and 73.3 ± 12.3 mL/min/1.73 m^2^ at weeks 12, 28, and 52, respectively.

**Table 3:** Treatment-emergent adverse events occurring in ≥2% of patients and adverse drug reactions occurring in ≥2 patients in any group (safety analysis set)

	Esaxerenone			
	Monotherapy (*n* = 245)	+ CCB (*n* = 59)	+ RAS inhibitor (*n* = 64)	Total (*N* = 368)
Any TEAE	160 (65.3)	46 (78.0)	47 (73.4)	253 (68.8)
Viral upper respiratory tract infection	54 (22.0)	21 (35.6)	26 (40.6)	101 (27.4)
Upper respiratory tract infection	8 (3.3)	0 (0.0)	0 (0.0)	8 (2.2)
Upper respiratory tract inflammation	8 (3.3)	3 (5.1)	4 (6.3)	15 (4.1)
Influenza	6 (2.4)	1 (1.7)	4 (6.3)	11 (3.0)
Bronchitis	8 (3.3)	0 (0.0)	0 (0.0)	8 (2.2)
Gastroenteritis	7 (2.9)	1 (1.7)	2 (3.1)	10 (2.7)
Dental caries	5 (2.0)	0 (0.0)	3 (4.7)	8 (2.2)
Diarrhea	7 (2.9)	1 (1.7)	2 (3.1)	10 (2.7)
Headache	9 (3.7)	0 (0.0)	0 (0.0)	9 (2.4)
Dermatitis contact	10 (4.1)	0 (0.0)	1 (1.6)	11 (3.0)
Arthralgia	3 (1.2)	5 (8.5)	1 (1.6)	9 (2.4)
Back pain	6 (2.4)	2 (3.4)	4 (6.3)	12 (3.3)
Renal impairment^a^	6 (2.4)	0 (0.0)	2 (3.1)	8 (2.2)
Hyperuricemia	3 (1.2)	6 (10.2)	0 (0.0)	9 (2.4)
Laboratory test	42 (17.1)	7 (11.9)	11 (17.2)	60 (16.3)
Serum K^+^ increased^a^	19 (7.8)	1 (1.7)	6 (9.4)	26 (7.1)
Any adverse drug reaction	45 (18.4)	14 (23.7)	12 (18.8)	71 (19.3)
Anemia	3 (1.2)	3 (5.1)	0 (0.0)	6 (1.6)
Hyperuricemia	1 (0.4)	6 (10.2)	0 (0.0)	7 (1.9)
Dizziness	0 (0.0)	1 (1.7)	1 (1.6)	2 (0.5)
Dizziness postural	1 (0.4)	0 (0.0)	1 (1.6)	2 (0.5)
Headache	2 (0.8)	0 (0.0)	0 (0.0)	2 (0.5)
Hepatic function abnormal	3 (1.2)	3 (5.1)	0 (0.0)	6 (1.6)
Renal impairment	4 (1.6)	0 (0.0)	1 (1.6)	5 (1.4)
Laboratory test	26 (10.6)	3 (5.1)	8 (12.5)	37 (10.1)
Serum K^+^ increased	18 (7.3)	1 (1.7)	6 (9.4)	25 (6.8)
Serum uric acid increased	2 (0.8)	0 (0.0)	0 (0.0)	2 (0.5)
Gamma-glutamyltransferase increased	2 (0.8)	0 (0.0)	0 (0.0)	2 (0.5)
Platelet count decreased	1 (0.4)	0 (0.0)	1 (1.6)	2 (0.5)
White blood cell count decreased	1 (0.4)	0 (0.0)	1 (1.6)	2 (0.5)
Lymphocyte percentage decreased	1 (0.4)	1 (1.7)	0 (0.0)	2 (0.5)
Serum K^+^ ≥5.5 mEq/L at any visit	14 (5.7)	2 (3.4)	4 (6.3)	20 (5.4)
Serum K^+^ ≥6.0 mEq/L or ≥5.5 mEq/L on two consecutive measurements	4 (1.6)	0 (0.0)	0 (0.0)	4 (1.1)
Serum K^+^ ≥6.0 mEq/L	2 (0.8)	0 (0.0)	0 (0.0)	2 (0.5)
Serum K^+^ ≥5.5 mEq/L on two consecutive measurements	3 (1.2)	0 (0.0)	0 (0.0)	3 (0.8)

Values are number of patients (%)

*CCB* calcium channel blocker, *RAS* renin–angiotensin system, *TEAE* treatment-emergent adverse events

^a^Renal impairment and serum K^+^ increased were defined as adverse events based on the judgement of the primary investigator as no clearly defined threshold values were available for these events

The most common treatment-emergent adverse event was viral upper respiratory tract infection, and the most common adverse drug reaction was increased serum K^+^ levels, which were observed in 7.3%, 1.7%, and 9.4% of patients in the esaxerenone monotherapy, CCB combination, and RAS inhibitor combination groups, respectively (Table 3). No patients in the combination therapy groups had hyperkalemia (serum K^+^ levels ≥6.0 mEq/L or ≥5.5 mEq/L on two consecutive measurements) during the treatment period; in the esaxerenone monotherapy group, two patients had serum K^+^ levels ≥6.0 mEq/L and three had serum K^+^ levels ≥5.5 mEq/L on two consecutive occasions (Table 3).

In one patient receiving esaxerenone 5 mg/day, the serum K^+^ level increased to 7.4 mEq/L from 3.6 mEq/L at baseline. This patient had no subjective symptoms related to hyperkalemia and exhibited no ECG changes. Subsequently, the primary investigator determined that in this case, elevated serum K^+^ occurred due to concurrent bacterial infection causing acute prostatitis and was not related to the study drug. In line with the discontinuation criteria, the study drug was discontinued, and the serum K^+^ level decreased to 4.7 mEq/L at the final follow-up.

Three patients withdrew from the study due to adverse events, including one each as a result of increased serum K^+^ level, dizziness, and angina pectoris.

## Discussion

This phase 3 clinical study was the first to investigate the long-term effects of esaxerenone, and the results demonstrate the long-term efficacy and safety of esaxerenone when given as a monotherapy or in combination with a CCB or RAS inhibitor in Japanese patients with essential hypertension. Antihypertensive effects on sitting BP were observed within 2 weeks after initiation of esaxerenone, and reductions in both sitting BP and 24-h ambulatory BP persisted for up to 28 or 52 weeks of treatment and were consistent across patient subgroups. The current study also showed that dose escalation of esaxerenone from 2.5 to 5 mg/day was feasible and that the majority of patients did not need additional antihypertensive therapy to achieve target BP.

These findings extend the result of a previous study that showed that esaxerenone has effective BP-lowering activity when administered over 12 weeks of therapy [40]. In that study performed in Japanese patients with essential hypertension, the effects of esaxerenone 2.5 mg/day on sitting and 24-h BP were noninferior to those of eplerenone 50 mg/day, and the BP-lowering effects of esaxerenone 5 mg/day were superior to those of the lower esaxerenone dosage [40]. In both the previous and current studies, the antihypertensive effects of esaxerenone persisted throughout the 24-h dosing window, and this may reflect the characteristics of this novel MR blocker, which include potent MR inhibition and a longer half-life than existing agents in this class [33], an effect that could be attributed to its nonsteroidal nature [41].

Reductions in sitting SBP during treatment with esaxerenone as a monotherapy or combination therapy ranged from 15 to 17 mmHg in week 12, from 17 to 20 mmHg in week 28, and from 21 to 24 mmHg in week 52. The magnitudes of these decreases are likely to be clinically significant [42]. The beneficial effects of esaxerenone therapy observed in the current study were also shown by the progressive and persistent reductions in natriuretic peptide levels during treatment, which were within the normal range and were consistent with other MR blockers [43]. Given the increasingly recognized role of MRs in the pathogenesis of cardiovascular and chronic kidney disease [44], current findings on esaxerenone are likely to have relevant clinical applications.

PAC and PRA were evaluated as indicators of the efficacy of esaxerenone for MR blockade because these parameters are increased by MR blockade. In the current study, PAC and PRA consistently increased in both the esaxerenone monotherapy and combination therapy groups until week 28 or week 52.

Long-term data from this study did not raise any new safety concerns for treatment with esaxerenone. Adverse event rates were similar when esaxerenone was given alone or in combination with other antihypertensive agents. Hyperkalemia was the most common adverse drug reaction. There was no tendency toward an increased risk of hyperkalemia with esaxerenone administration, regardless of treatment duration. There were no cases of hyperkalemia in the esaxerenone combination therapy groups, even when esaxerenone was combined with a RAS inhibitor, but two patients receiving esaxerenone monotherapy had serum K^+^ levels ≥6.0 mEq/L, and three had serum K^+^ levels ≥5.5 mEq/L on two consecutive occasions (overall rate 1.1%). The serum K^+^ level increased within 2 weeks after the initiation of esaxerenone, but no trend toward an increase in serum K^+^ levels was observed at the time of esaxerenone dose escalation. Because the dose escalation criteria included other factors in addition to the serum K^+^ concentration, it was not possible to specify the exact reason for nonescalation in each case. Although our findings do not suggest a risk of increased serum K^+^ levels when esaxerenone is administered in combination with a RAS inhibitor, it is necessary to carefully monitor serum K^+^ levels to minimize the risk of hyperkalemia when using this combination treatment. Additionally, the increase in serum K^+^ levels occurred mostly up to week 28, with no further consistent increases observed during long-term treatment. Based on these results, the increases in serum K^+^ levels observed during treatment with esaxerenone appear to be clinically acceptable, and a long-term therapy with this novel MR blocker is possible with appropriate monitoring of serum K^+^ levels.

### Study limitations

The most important limitations of this study relate to its design. Patients were not randomized to different treatment groups, all patients and investigators were aware of the treatment received (i.e., no blinding), and there was no standard therapy comparator arm. Additionally, the patient population was comprised exclusively of patients from Japan, meaning that the generalizability of these findings to other populations may be limited. Therefore, the results should be interpreted with caution and taken as exploratory.

## Conclusion

The administration of esaxerenone treatment over 1 year was associated with sustained and stable antihypertensive effects in patients with essential hypertension whether provided as a monotherapy or when given in combination with a CCB or RAS inhibitor. The antihypertensive efficacy of esaxerenone and its good safety profile suggest that this novel MR blocker may be a promising treatment option for patients with hypertension and could be used in combination therapy regimens to achieve substantial BP reductions and a good BP control.

## upplementary information

Supplementary Table1
Supplementary Table2
Supplementary Figure1
Supplementary Figure2A
Supplementary Figure2B
Supplementary Figure3
Supplementary Figure4
Supplementary Figure5
Supplementary Figure6
Supplementary Information
