# Supplementary material for: Long-term phase 3 study of esaxerenone as mono or combination therapy with other antihypertensive drugs in patients with essential hypertension
Source: Hypertens Res. 2019 Sep 25;42(12):1932–41. doi: 10.1038/s41440-019-0314-7 (PMC8076031; doi:10.1038/s41440-019-0314-7)
Supplement: Supplementary file 1 — Supplementary Table1 [file 41440_2019_314_MOESM1_ESM.docx]

## Supplementary table 1. Mean change from baseline in sitting SBP and DBP in the monotherapy group without any additional antihypertensive medications after Week 12

|  | Week 12 | | | Week 28 | | | Week 52 | | |
| --- | --- | --- | --- | --- | --- | --- | --- | --- | --- |
|  | *n* | SBP | DBP | *n* | SBP | DBP | *n* | SBP | DBP |
| Change in blood pressure, mmHg | 156 | −17.1 ± 12.04 | −8.0 ± 7.40 | 156 | −18.0 ± 11.66 | −8.9 ± 7.02 | 51 | −19.0± 12.97 | −10.2 ± 7.91 |

Values are mean ± SD.

Abbreviations: DBP, diastolic blood pressure; SBP, systolic blood pressure.
