# Supplementary material for: Long-term phase 3 study of esaxerenone as mono or combination therapy with other antihypertensive drugs in patients with essential hypertension
Source: Hypertens Res. 2019 Sep 25;42(12):1932–41. doi: 10.1038/s41440-019-0314-7 (PMC8076031; doi:10.1038/s41440-019-0314-7)
Supplement: Supplementary file 2 — Supplementary Table2 [file 41440_2019_314_MOESM2_ESM.docx]

## Supplementary table 2. Subgroup analysis of the least-squares mean change from baseline in sitting SBP and DBP

| Change in blood pressure, mmHg  Mean (95% CI) | Week 12 (*n*=368) | | | Week 28 (*n*=368) | | | | | | Week 52 (*n*=147) | | | | | |
| --- | --- | --- | --- | --- | --- | --- | --- | --- | --- | --- | --- | --- | --- | --- | --- |
|  | *n* | SBP | DBP | *n* | | SBP | | DBP | | *n* | | SBP | | DBP | |
| Age |  |  |  |  | |  | |  | |  | |  | |  | |
| <65 years | 290 | −14.9  (−16.3, −13.6) | −7.2  (−8.0, −6.3) | 290 | −18.1  (−19.5, −16.7) | | −9.6  (−10.5, −8.8) | | 113 | | −21.7  (−23.8, −19.5) | | −11.5  (−12.8, −10.2) | |  |
| ≥65 years |  | −20.6  (−23.2, −18.0) | −9.5  (−11.0, −7.9) | 78 | −22.0  (−24.7, −19.3) | | −11.1  (−12.6, −9.6) | | 34 | | −27.7  (−32.1, −23.3) | | −15.7  (−17.9, −13.5) | |  |
| Baseline sitting SBP |  |  |  |  |  | |  | |  | |  | |  | |  |
| <160 mmHg | 250 | −13.7  (−15.0, −12.4) | −7.3  (−8.2, −6.4) | 250 | | −15.3  (−16.6,−14.1) | | −8.8  (−9.7, −8.0) | | 102 | | −19.3  (−21.5, −17.2) | | −11.2  (−12.6, −9.8) | |
| ≥160 mmHg | 118 | −21.2  (−23.6, −18.8) | −8.5  (−9.9, −7.1) | 118 | | −26.6  (−28.9, −24.3) | | −12.3  (−13.7, −11.0) | | 45 | | −31.5  (−34.5, −28.5) | | −15.4  (−17.2, −13.5) | |
| Hypertension grade |  |  |  |  | |  | |  | |  | |  | |  | |
| I | 176 | −13.4  (−14.9, −11.9) | −6.4  (−7.4, −5.4) | 176 | | −15.2  (−16.7, −13.7) | | −8.0  (−9.0, −7.0) | | 78 | | −19.1  (−21.6, −16.6) | | −10.7  (−12.2, −9.1) | |
| II | 192 | −18.7  (−20.4, −16.9) | −8.8  (−9.9, −7.7) | 192 | | −22.3  (−24.2, −20.5) | | −11.7  (−12.7, −10.7) | | 69 | | −27.6  (−30.4, −24.7) | | −14.5  (−16.1, −12.8) | |
| Comorbid type 2 diabetes | |  |  |  | |  | |  | |  | |  | |  | |
| No | 301 | −16.4  (−17.7, −15.0) | −7.7  (−8.5, −6.8) | 301 | | −18.8  (−20.1, −17.4) | | −9.8  (−10.6, −8.9) | | 116 | | −21.8  (−23.9, −19.7) | | −11.9  (−13.1, −10.7) | |
| Yes | 67 | −15.1  (−17.8, −12.3) | −7.6  (−9.3, −5.9) | 67 | | −19.7  (−22.6, −16.7) | | −10.8  (−12.6, −9.1) | | 31 | | −27.8  (−32.6, −23.0) | | −14.6  (−17.8, −11.4) | |

Abbreviations: CI, confidence interval; DBP, diastolic blood pressure; SBP, systolic blood pressure.
