# Supplementary figures and images for: Long-term phase 3 study of esaxerenone as mono or combination therapy with other antihypertensive drugs in patients with essential hypertension
Source: Hypertens Res. 2019 Sep 25;42(12):1932–41. doi: 10.1038/s41440-019-0314-7 (PMC8076031; doi:10.1038/s41440-019-0314-7)

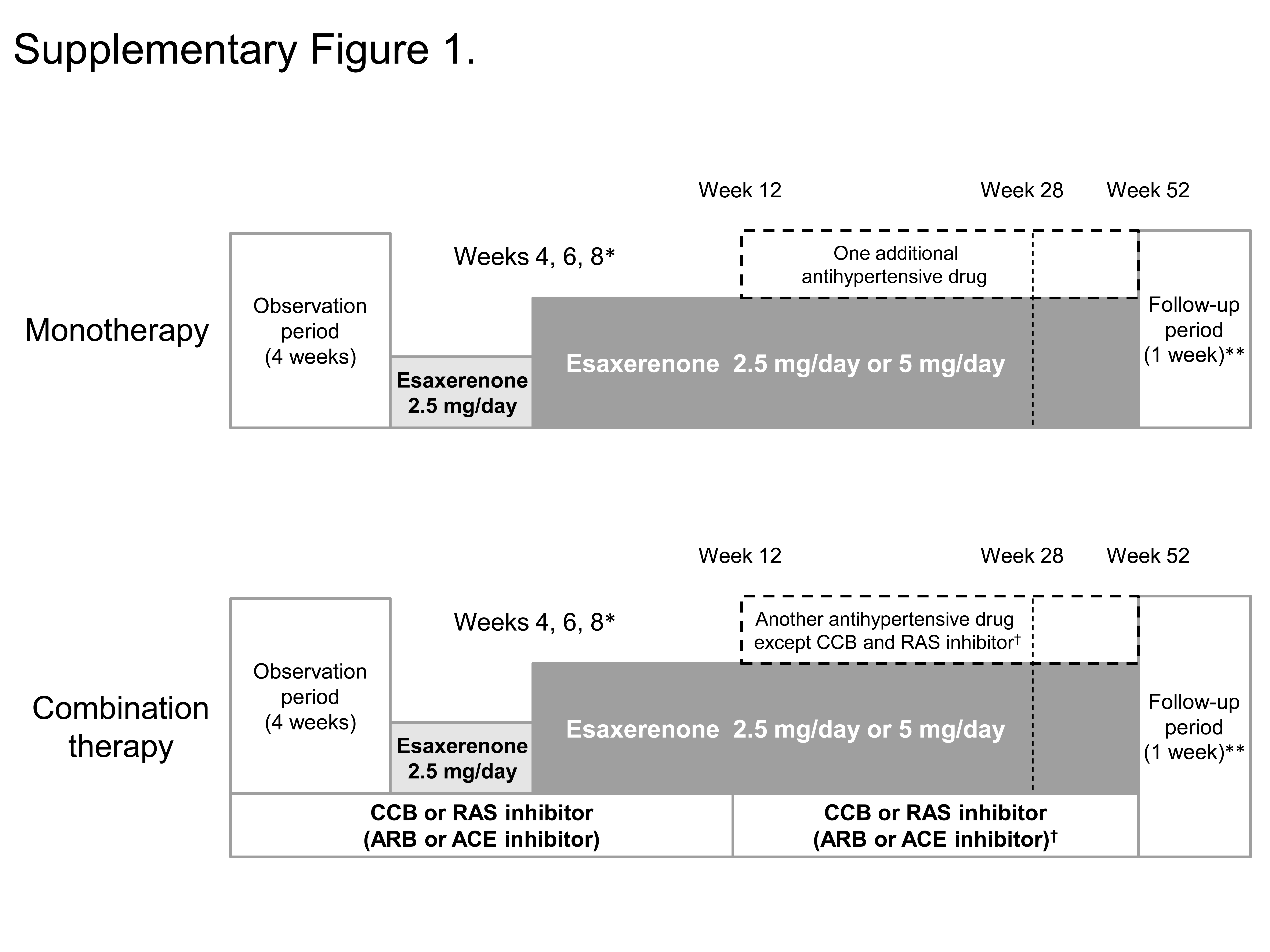

Supplement: Supplementary file 3 — Supplementary Figure1 [file 41440_2019_314_MOESM3_ESM.tif]

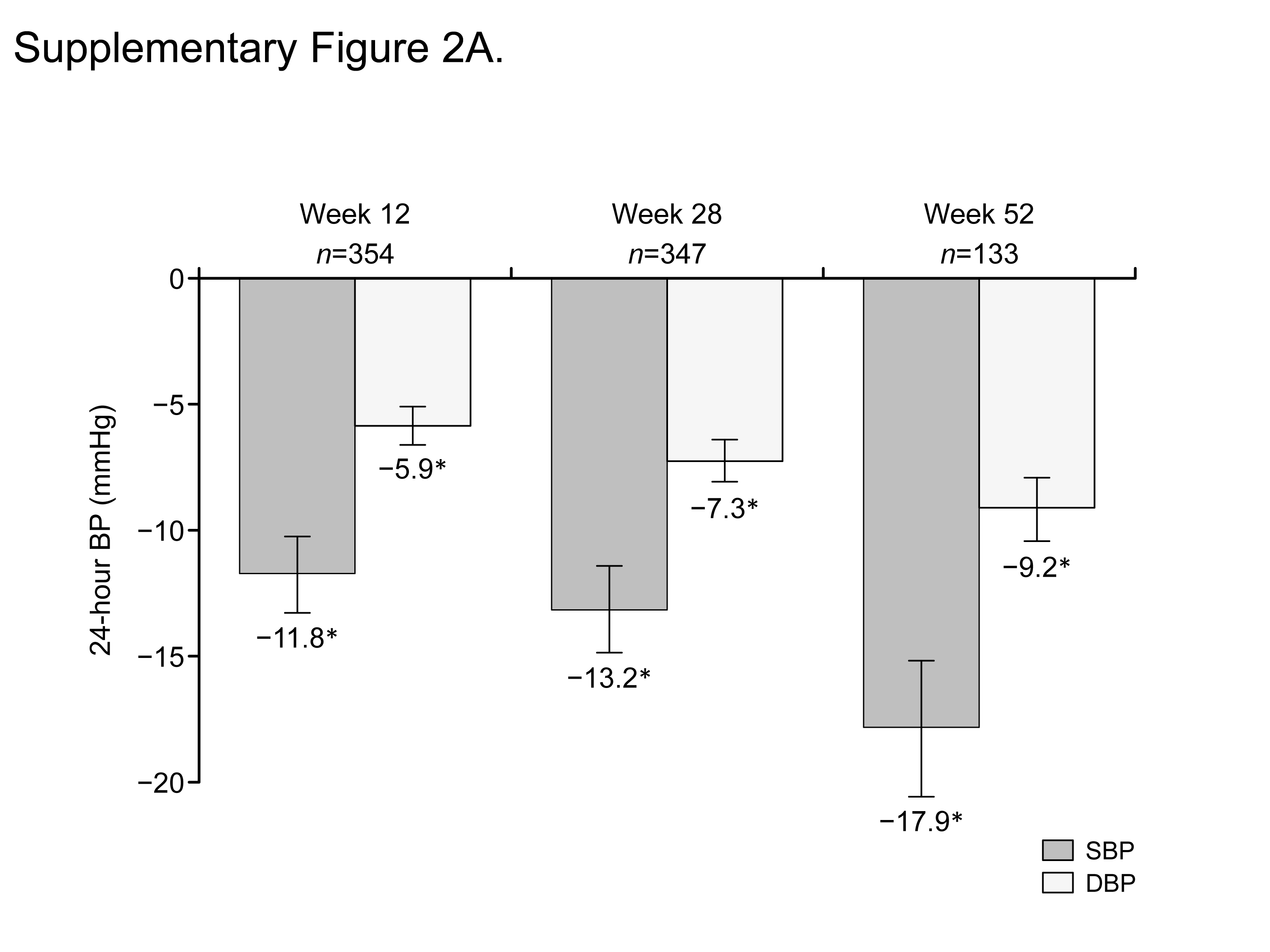

Supplement: Supplementary file 4 — Supplementary Figure2A [file 41440_2019_314_MOESM4_ESM.tif]

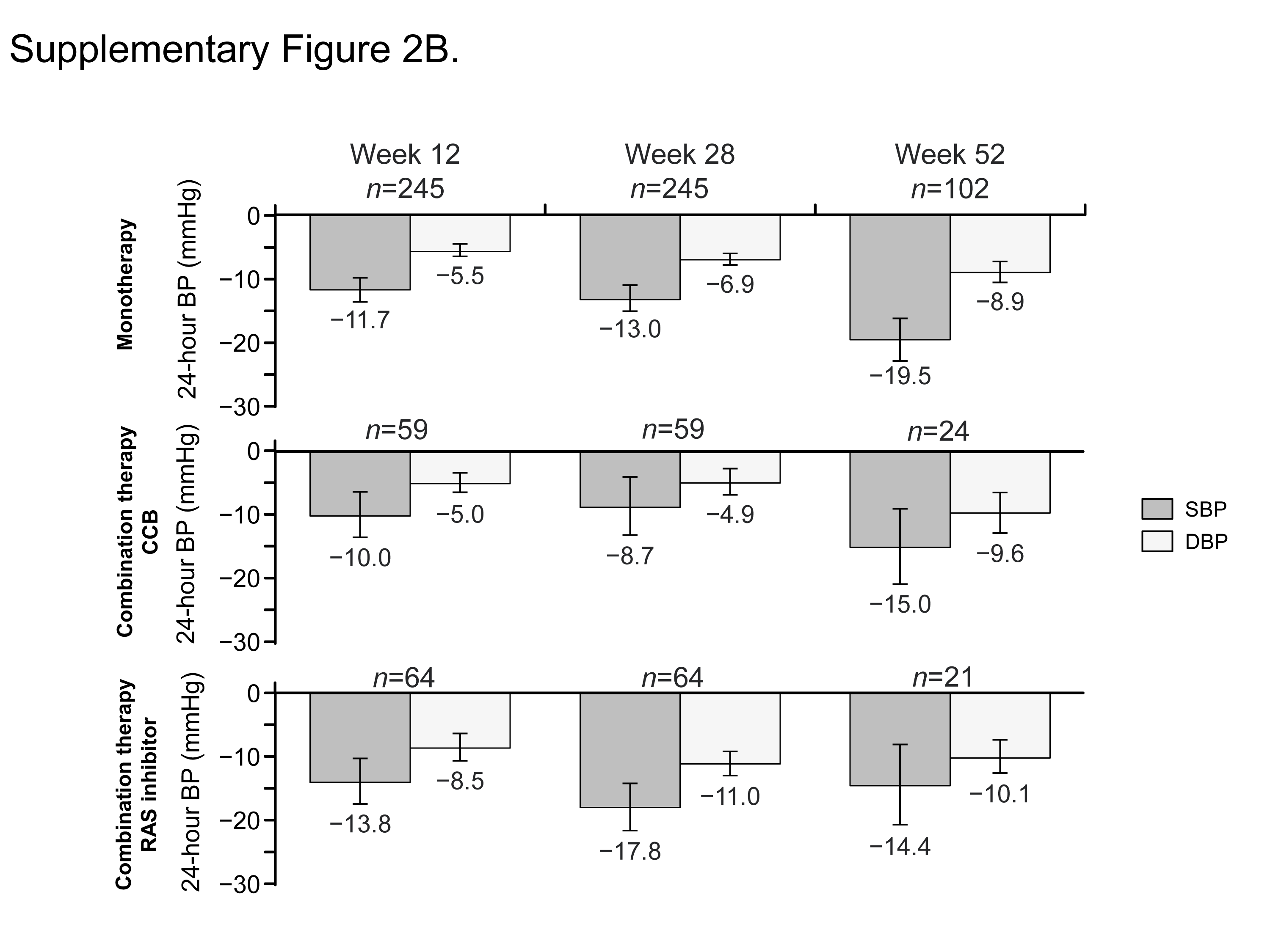

Supplement: Supplementary file 5 — Supplementary Figure2B [file 41440_2019_314_MOESM5_ESM.tif]

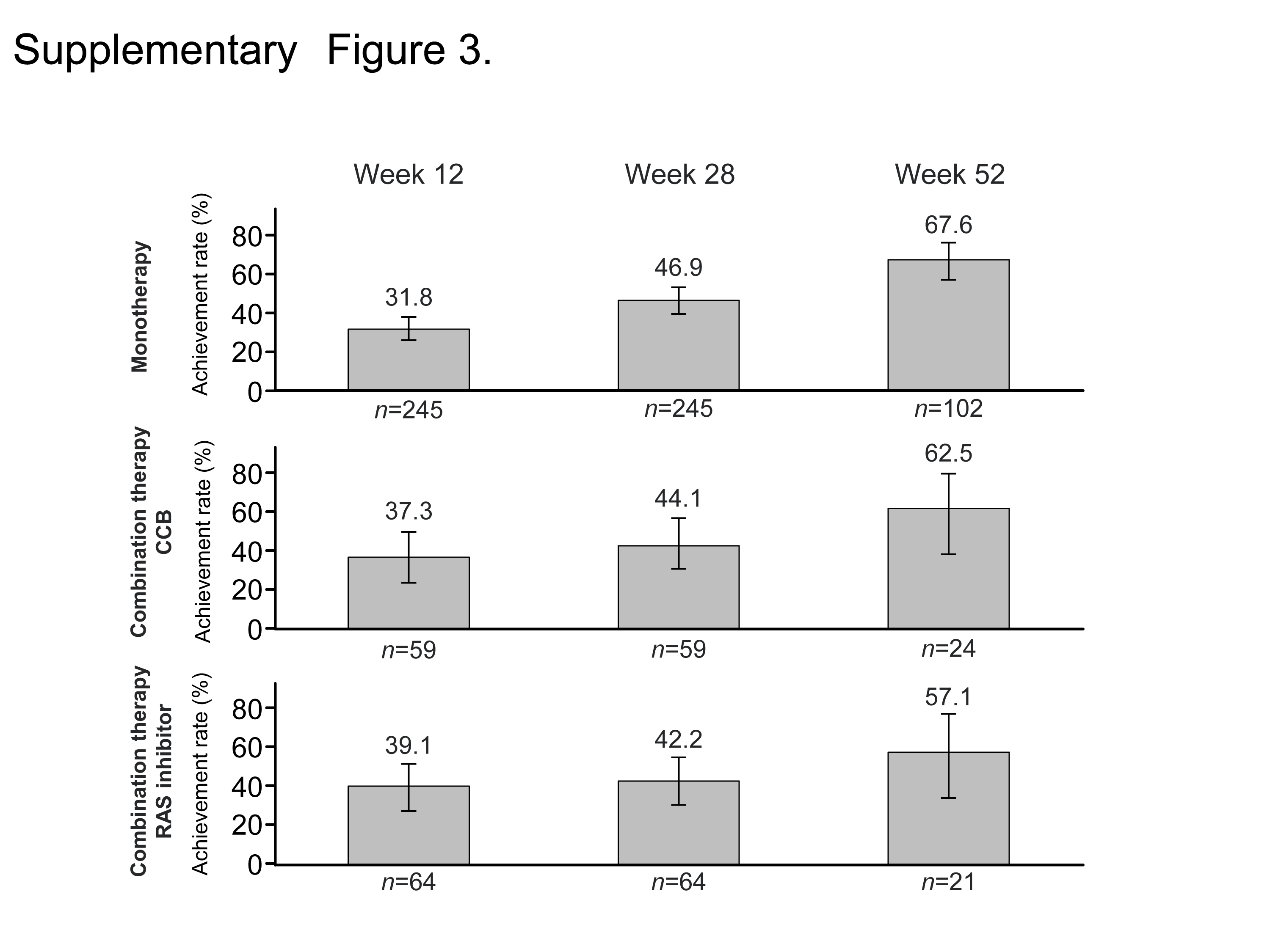

Supplement: Supplementary file 6 — Supplementary Figure3 [file 41440_2019_314_MOESM6_ESM.tif]

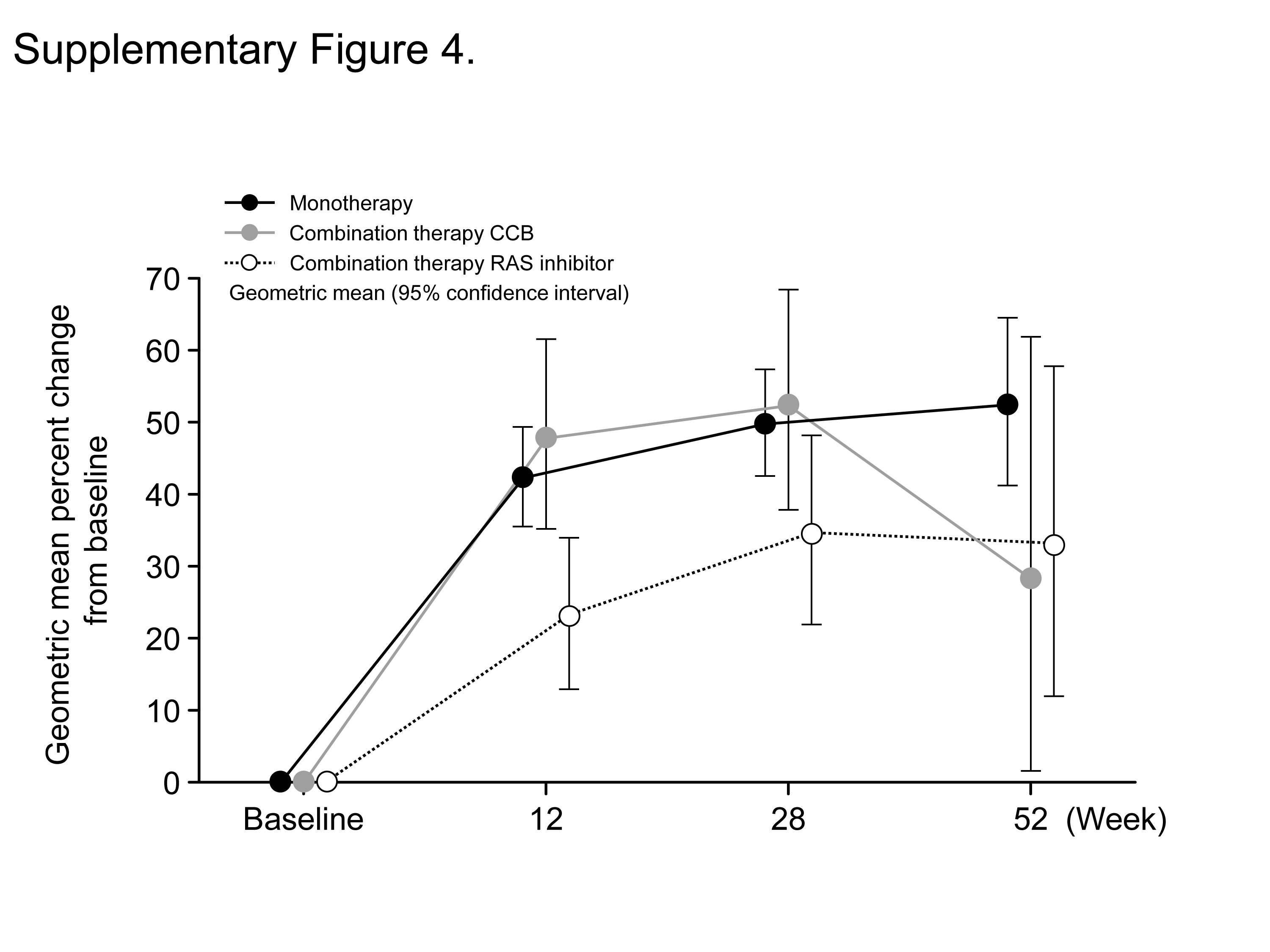

Supplement: Supplementary file 7 — Supplementary Figure4 [file 41440_2019_314_MOESM7_ESM.tif]

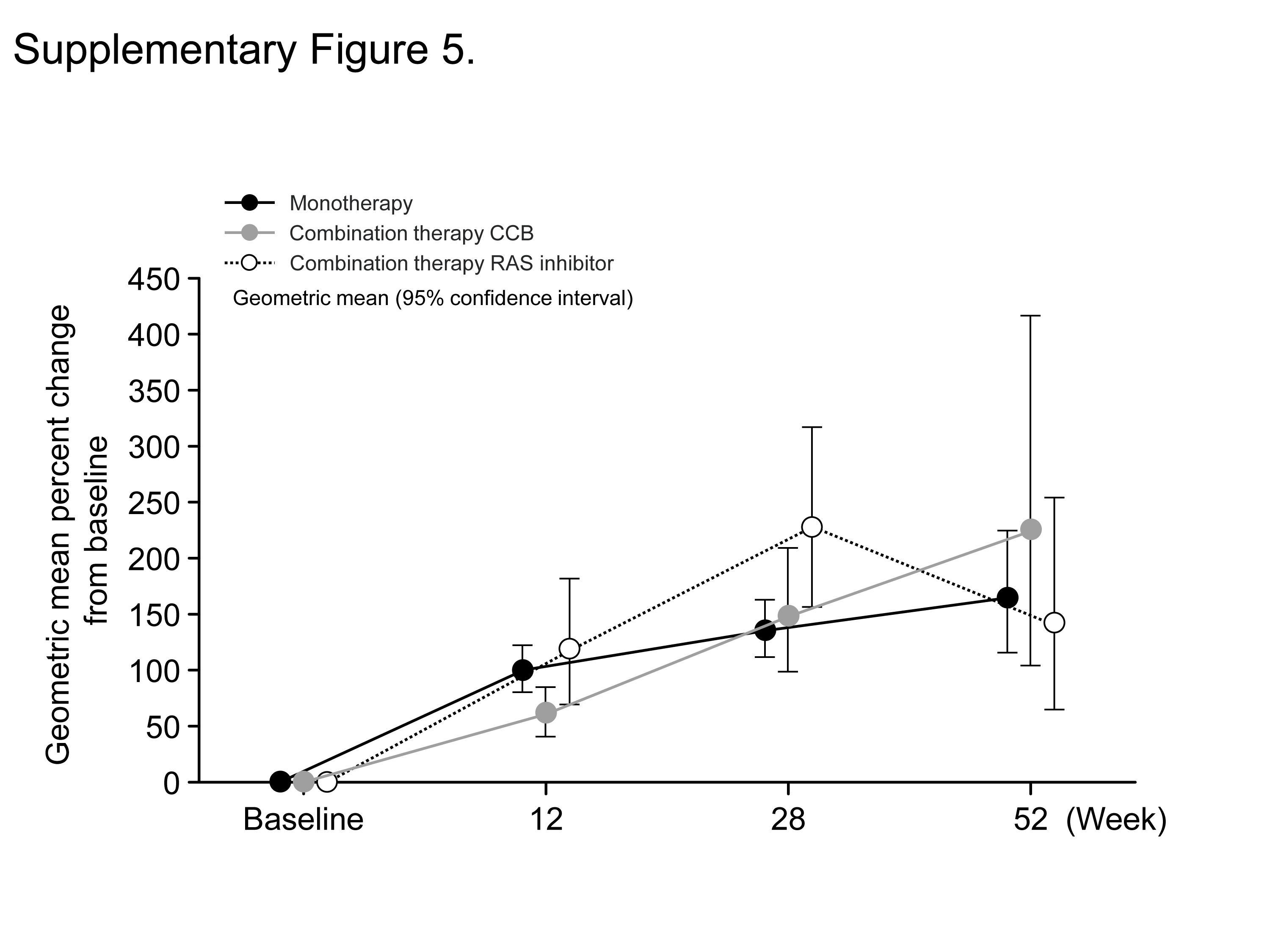

Supplement: Supplementary file 8 — Supplementary Figure5 [file 41440_2019_314_MOESM8_ESM.tif]

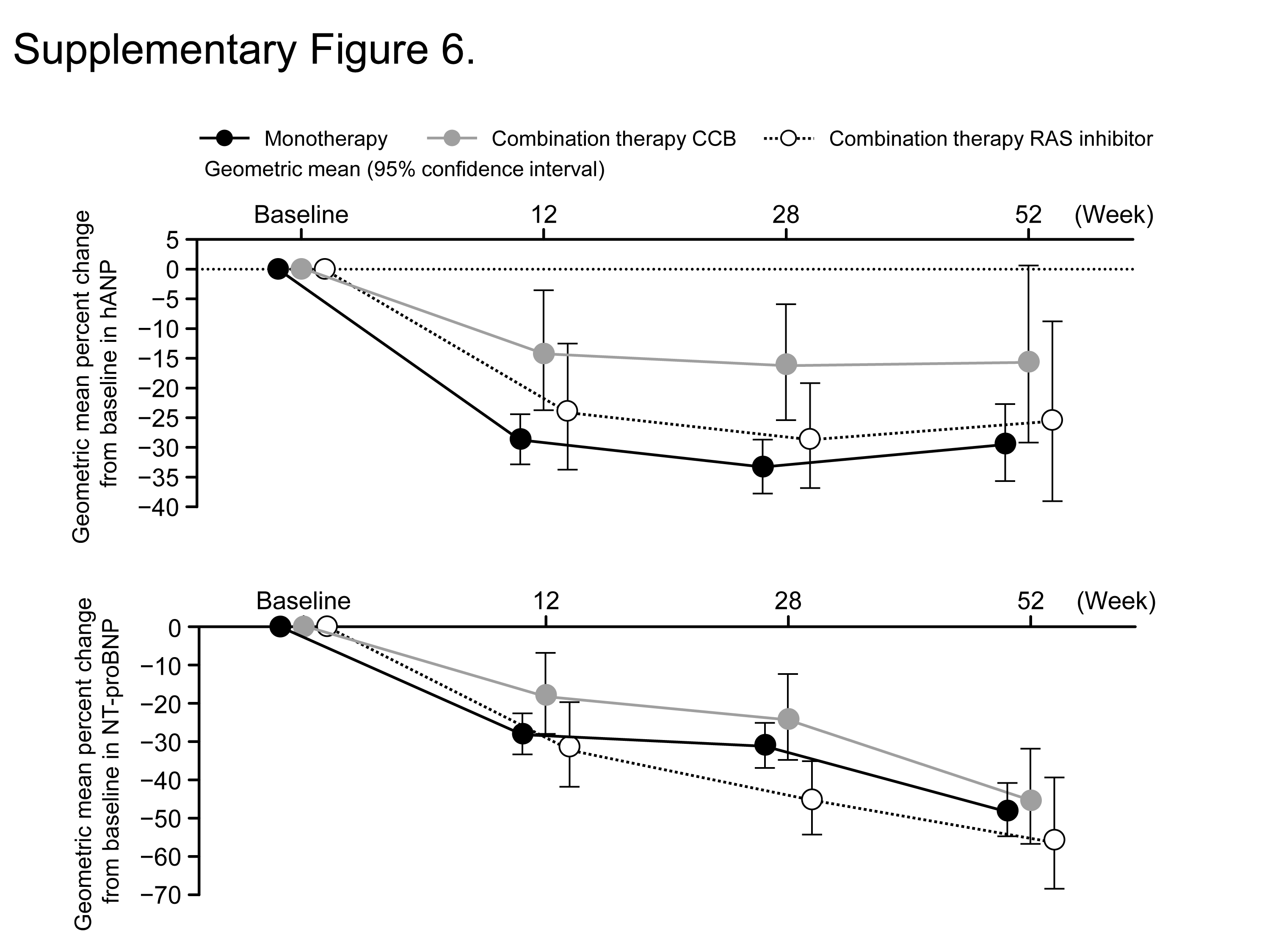

Supplement: Supplementary file 9 — Supplementary Figure6 [file 41440_2019_314_MOESM9_ESM.tif]
