# Supplementary material for: Long-term phase 3 study of esaxerenone as mono or combination therapy with other antihypertensive drugs in patients with essential hypertension
Source: Hypertens Res. 2019 Sep 25;42(12):1932–41. doi: 10.1038/s41440-019-0314-7 (PMC8076031; doi:10.1038/s41440-019-0314-7)
Supplement: Supplementary file 10 — Supplementary Information [file 41440_2019_314_MOESM10_ESM.docx]

# Supplementary Information

## Supplementary table 1 Mean change from baseline in sitting SBP and DBP in the monotherapy group treated without any additional antihypertensive medications after week 12.

## Supplementary table 2. Subgroup analysis of the least-squares mean change from baseline in sitting SBP and DBP.

**Supplementary figure 1.** Study design.

**Supplementary figure 2.** Mean change from baseline in 24-hour BP (SBP/DBP) for each treatment group.

**Supplementary figure 3.** Target blood pressure achievement rate (full analysis set). Target sitting blood pressure criteria: <140/90 mmHg. Data are shown as the mean (95% confidence interval).

**Supplementary figure 4.** Geometric mean percent change from baseline in plasma aldosterone concentration over time.

**Supplementary figure 5.** Geometric mean percent change from baseline in plasma renin activity over time.

**Supplementary figure 6**. Geometric mean percent change from baseline in human atrial natriuretic polypeptide (hANP) and N-terminal pro B-type brain natriuretic peptide (NT-proBNP) over time.

# SUPPLEMENTARY FIGURE LEGENDS

**Supplementary figure 1.** Study design.

*The esaxerenone dosage was titrated at weeks 4, 6, and 8 based on the dose escalation criteria. **Follow-up was performed at 1 week after Week 28 or Week 52 with appropriate antihypertensive treatment.

^†^Dose escalation of the baseline CCB or RAS inhibitor or additional use of only one additional concomitant antihypertensive drug (CCB, thiazide diuretic, or RAS inhibitor) other than a basic antihypertensive drug was permitted at week 12.

ACE-I, angiotensin-converting enzyme inhibitor; ARB, angiotensin II receptor blocker; CCB, calcium channel blocker; RAS, renin–angiotensin system

**Supplementary figure 2.** Mean change from baseline in 24-hour BP (SBP/DBP) for each treatment group: (**A**) all patients and (**B**) monotherapy and combination therapy (full analysis set). Data are shown as the mean (95% confidence interval); paired *t*-test. **P*<0.0001 vs baseline.

Abbreviations: BP, blood pressure; CCB, calcium channel blocker; DBP, diastolic BP; RAS, renin–angiotensin system; SBP, systolic BP.

**Supplementary figure 3.** Target blood pressure achievement rate (full analysis set). Target sitting blood pressure criteria: <140/90 mmHg. Data are shown as the mean (95% confidence interval).

Abbreviations: CCB, calcium channel blocker; RAS, renin–angiotensin system.

**Supplementary figure 4.** Geometric mean percent change from baseline in plasma aldosterone concentration over time.

Abbreviations: CCB, calcium channel blocker; RAS, renin–angiotensin system.

**Supplementary figure 5.** Geometric mean percent change from baseline in plasma renin activity over time.

Abbreviations: CCB, calcium channel blocker; RAS, renin–angiotensin system.

**Supplementary figure 6**. Geometric mean percent change from baseline in human atrial natriuretic polypeptide (hANP) and N-terminal pro B-type brain natriuretic peptide (NT-proBNP) over time.

Abbreviations: CCB, calcium channel blocker; RAS, renin–angiotensin system.
